# Exophthalmos and hemiheadache caused by osteoma in the greater wing of sphenoid bone: an extremely rare case report

**DOI:** 10.1097/MS9.0000000000000504

**Published:** 2023-04-01

**Authors:** Yamama Tawashi, Kenana Tawashi, Tasneem Beski, Khaleel Alhakeem

**Affiliations:** a Faculty of Medicine, Hama University; b Neurosurgery Department, Hama National Hospital, Hama, Syria

**Keywords:** case report, exophthalmos, greater wing, osteoma, sphenoid bone

## Abstract

Osteoma is the most common benign tumor of the nose and paranasal sinuses. It is usually asymptomatic and diagnosed accidentally. In our case, the tumor formed in an unusual location and led to unexpected symptoms, which created a big challenge in diagnosis and treatment.

**Case presentation:**

A 53-year-old woman complained of hemiheadache, exophthalmos in the right eye, and limitation in lateral eye movements progressing to diplopia in the past 2 months. The physical examination of the rest systems was unremarkable. The radiological investigations revealed a hyperdense lesion arising from the right greater wing of the sphenoid bone and compressed on the orbit’s components and eye muscles, which caused proptosis. The radiological findings suggested osteoma and the tumor was excised by craniotomy. The patient gets rid of the symptoms and the follow-up for 6 months was uneventful.

**Clinical discussion:**

Even hemiheadache, exophthalmos, limitation in eye movements, and diplopia are unfamiliar findings in osteoma, they may be its manifestations. Also, MRI is used as a diagnostic method with computed tomography scan in intracranial osteoma. These cases are treated by craniotomy.

**Conclusions:**

Even though osteoma is a benign tumor, it may form in unusual locations and cause unexpected symptoms. So, it should be a differential diagnosis in skull bony tumors. Also, it should be treated when exists in sensitive places to avoid irreversible outcomes.

## Introduction

Highlights

- Osteoma is often asymptomatic and diagnosed accidentally, but when it locates in a sensitive location it may cause severe symptoms.
- MRI is an important method in the diagnosis and treatment of intracranial osteoma.
- Osteoma should be a differential diagnosis in skull bony tumors.

Osteomas are primary benign slow-growing bony tumors. They develop in the fourth to sixth decades, with a male preponderance^1^,^2^. The most affected sinus is the frontal (71.8%), followed by the ethmoidal sinus (16.9%) and maxillary sinus (6.4%), while the sphenoid sinus is an unusual location (4.9%)^3^. The osteomas can be classified according to their location in the skull into skull vault, skull base, dural, and intraparenchymal osteoma^1^. Osteoma is diagnosed in most cases by radiological investigations, which are indicated for other reasons because it is often asymptomatic^4^. Symptoms if they exist depend on location, size, the direction of growth, and extension^3^,^5^. Asymptomatic osteoma does not need treatment, while symptomatic one should be excised^4^. We discuss in this paper an extremely rare location of osteoma with unusual manifestations, which makes the diagnosis and treatment vary hard. This manuscript has been reported in line with SCARE 2020 criteria^6^.

## Case presentation

**Figure 1:**
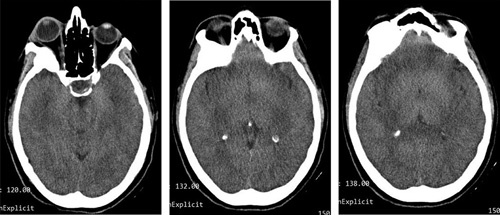
The head computed tomography scan revealed a hyperdense lesion arising from the right greater wing of the sphenoid bone and compressed on the orbit’s components and eye muscles, which caused proptosis.

**Figure 2:**
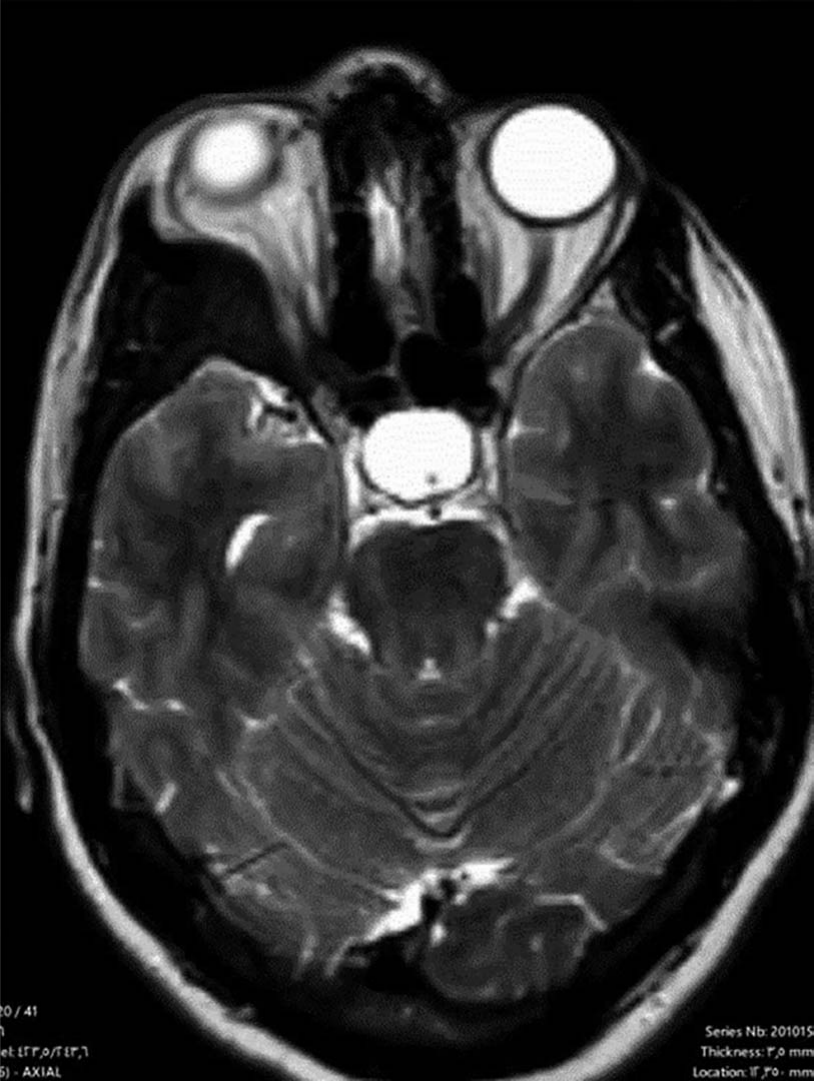
The MRI revealed a lesion with a low signal on T2-weighted in the lateral wall of the orbit compresses the rectus lateralis muscle on the right side, causing proptosis with light pressure on the anterior part of the right temporal lobe.

**Figure 3:**
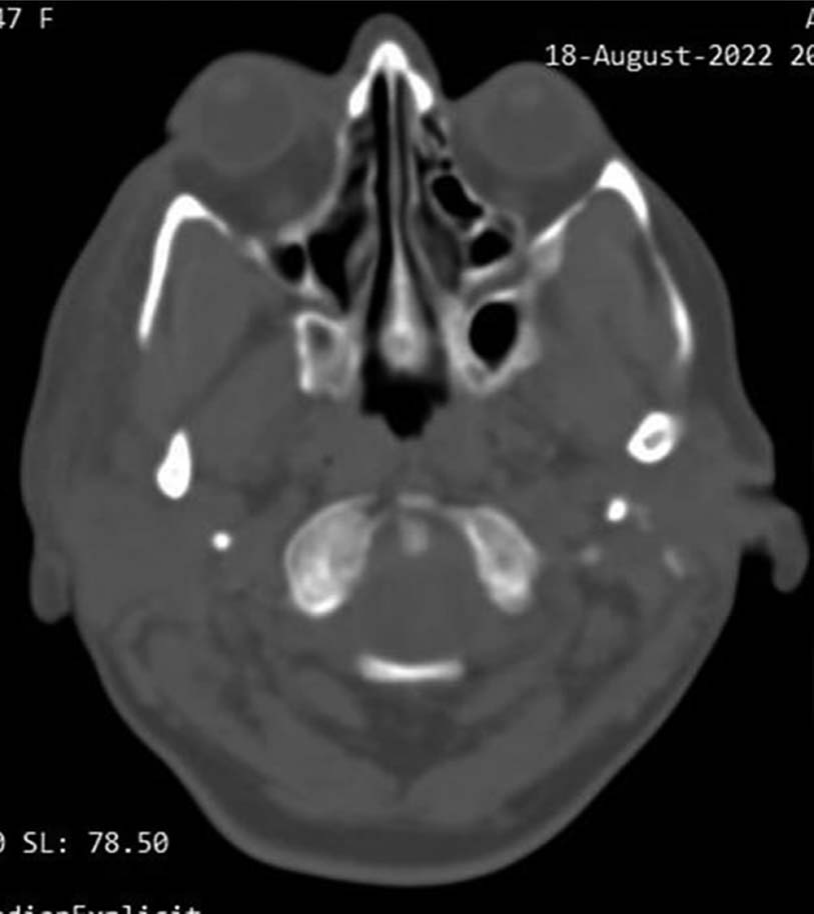
The computed tomography scan after 2 weeks revealed normal findings with no residues, and the proptosis significantly decreased.

A 53-year-old woman was admitted to the Neurosurgery Department, referred by an ophthalmology physician, complaining of hemiheadache, progressive exophthalmos of the right eye for 2 years, and developed restricted eye movements in the lateral side, which led to diplopia during the past 2 months. She had no medical, surgical, allergic, drug, or familial history. The patient was aware and responsive and the physical examination was unremarkable. All the laboratory investigations were within normal. The head computed tomography (CT) scan revealed a hyperdense lesion arising from the right greater wing of the sphenoid bone and compressed on the orbit’s components and eye muscles, which caused proptosis (Fig. 1). The MRI revealed a lesion with a low signal on T2-weighted in the lateral wall of the orbit compressed the rectus lateralis muscle on the right eye, causing proptosis with light pressure on the anterior part of the right temporal lobe (Fig. 2). These findings suggested osteoma even though it is not familiar in this location and classified meningioma as differential diagnoses. The surgical excision was indicated and written consent from the patient was taken. Medical consultations before surgery were taken. The tumor was removed by craniotomy under general anesthesia by a specialist in neurosurgery. Before the surgery, there was a differential diagnosis of calcified meningioma. But during the surgery, the tumor was a thickening of the sphenoid bone without any adhesion to the meninges, or invasion of neighboring muscles. The edges of the tumor were clearly defined and regular, without any signs of malignancy. The operation went according to plan except that we were forced to transfer blood to improve the hemoglobin after unexpected little bleeding. After surgery, the patient was given three letters of physiological serum, prophylaxis antibiotics, analgesics, and NSAIDs. The physical examination after 2 days of the surgery was normal, the eye’s movements returned normal, the exophthalmos significantly decreased, and the pupils were reflexive and symmetrical. The laboratory investigations were normal except for low hemoglobin (8 mg/dl); the patient was discharged. Histopathology examination was compatible with osteoma. The CT scan after 2 weeks revealed normal findings with no residues, and the proptosis significantly decreased (Fig. 3). The patient was followed up for 6 months physically and radiologically without any signs of recurrence.

## Discussion

Skull’s bony tumors from 0.8 to 1% of all bone tumors. Osteoma is a primary benign bony tumor, which considering the most primary benign tumor of the nose and paranasal sinuses^1^,^3^. In addition to its usual location, it can be found in rare places like the lateral pterygoid plate of the sphenoid bone, subdural or subarachnoid space, tongue base, and greater wing of the sphenoid bone as in our case^7^–^9^. It can be peripheral (originating from the periosteum) or central (originating from the endosteum) or extraskeletal, which forms in soft tissues. Histopathology, osteoma is divided into compact (ivory), and trabecular (mature) osteomas. Ivory osteoma consists of mature lamellar bone with a little amount of marrow space, Haversian canals, and fibrous structure. While mature osteoma consists of cancellous trabecular bone, fibrofatty marrow, and osteoblasts^4^,^5^. Many hypotheses have been suggested to explain the etiology of osteoma including developmental, traumatic, and infective. Developmental theory suggests that osteoma develops from the fusion area between frontal and ethmoidal bones. Traumatic theory suggests that osteoma develops as a reaction to trauma, while infective theory postulates that osteoma is an osteoblast induced by an infection. In addition, multiple osteomas are the manifestation of a hereditary autosomal dominant case, Gardner syndrome which is diagnosed by multiple osteomas, soft tissue tumors, and colonic polyposis^2^. We keep many differential diagnoses in our mind until the terminal diagnosis was made by histopathology such as osteogenesis imperfecta, ossifying fibroma, bone infarction, endochondroma, fibrous dysplasia, low-grade osteogenic sarcoma, and calcified meningioma^10^. The location of the tumor with the radiological and clinical findings in our case also suggested en-plaque meningioma, which is a rare pattern of meningioma that pervades the dura and the bone. En-plaque meningioma’s characteristics that recommend it include its location in the sphenoid wing, growing alone on the bone border, female preponderance, the age of presentation in the fifth decade, and clinical symptoms like proptosis and headache^11^. Osteoma is often asymptomatic. When it is symptomatic, the most common one is headache, which is resulted from the tumor itself, or sinusitis caused by obstruction of the sinus drainage^2^. The symptoms in special cases such as sphenoid sinus and orbital osteomas include headache, proptosis, chemosis, and diplopia if the eye’s muscles are affected, epiphora if the nasolacrimal duct is obstructed, visual disturbances, and in rare cases complete blindness and neurological signs^2^,^3^. Osteoma is often diagnosed by radiological investigations performed for other indications^5^. CT is the cornerstone in diagnosing osteoma, it helps in the evaluation of the size, location, extension, and site origin of the tumor^2^,^10^. In addition, it is used for planning the surgery and follow-up^5^. Osteoma appears on CT scans as a round or oval high-density lesion, with clear and smooth margins^4^. MRI is used in special cases like intracranial or intraorbital extension, many differential diagnoses, and complications related to the tumor, all these indications get together in our case^4^,^5^. The main treatment of asymptomatic osteoma is observation and regular follow-up because of the slow growth rate, whereas surgery is indicated in some situations like large osteoma, osteoma extending more than 50% of the frontal sinus, symptomatic osteoma, and cosmetic reasons. Sphenoid osteoma is the only exception; some studies encourage treating it even if it is asymptomatic because of the risk of blindness, while others encourage watchful observation in asymptomatic cases because until now there is no specific protocol to deal with sphenoid osteoma^3^,^5^,^12^. Radical excision is the basic procedure in treatment; it should be done with the least invasive technique and the best cosmetic results^4^. In our case and because of the intracranial extension, symptoms, and signs we resorted to surgery where we excised the whole tumor.

## Conclusions

Even though osteoma is a slow-growing benign tumor, it can form in unusual locations and cause unexcepted manifestations. So, it should be a differential diagnosis in skull bony tumors. Also, regular follow-up is a necessary need, especially in the sensitive location where the tumor may cause severe complications and in rare cases irreversible consequences. These situations demand more investigations like MRI and unfamiliar treatments like craniotomy. This paper highlights many questions about the hypothesis that explain the etiology of osteoma and encourage more studies to establish the basic procedures for dealing with these rare cases.

## Ethical approval

Ethical approval was not required.

## Consent

Written informed consent was obtained from the patient for the publication of this case report and accompanying images. A copy of the written consent is available for review by the Editor-in-Chief of this journal on request.

## Sources of funding

No funding is required.

## Author contribution

Y.T. collected the patient’s data and drafted the manuscript. K.T. drafted the manuscript. T.B. drafted the manuscript. K.A. collected the patient’s data, performed the procedure, revised the manuscript, and supervised the study.

## Conflicts of interest disclosure

The authors have no confilict of interest.

## Research registration unique identifying number (UIN)

None.

## Guarantor

Yamama Tawashi.

## Provenance and peer review

Not commissioned, externally peer-reviewed.
